# Biomarkers for risk-based treatment modifications for CNS germ cell tumors: Updates on biological underpinnings, clinical trials, and future directions

**DOI:** 10.3389/fonc.2022.982608

**Published:** 2022-09-05

**Authors:** Hirokazu Takami, Koichi Ichimura

**Affiliations:** ^1^ Department of Neurosurgery, The University of Tokyo Hospital, Tokyo, Japan; ^2^ Department of Brain Disease Translational Research, Juntendo University Faculty of Medicine, Tokyo, Japan

**Keywords:** germ cell tumor, germinoma, non-germinomatous, biomarker, clinical trial

## Abstract

CNS germ cell tumors (GCTs) preferentially occur in pediatric and adolescent patients. GCTs are located predominantly in the neurohypophysis and the pineal gland. Histopathologically, GCTs are broadly classified into germinomas and non-germinomatous GCTs (NGGCTs). In general, germinoma responds well to chemotherapy and radiation therapy, with a 10-year overall survival (OS) rate of approximately 90%. In contrast, NGGCTs have a less favorable prognosis, with a five-year OS of approximately 70%. Germinomas are typically treated with platinum-based chemotherapy and whole-ventricular radiation therapy, while mature teratomas can be surgically cured. Other NGGCTs require intensive chemotherapy with radiation therapy, including whole brain or craniospinal irradiation, depending on the dissemination status and protocols. Long-term treatment-related sequelae, including secondary neoplasms and cerebrovascular events, have been well recognized. These late effects have a tremendous impact in later life, especially since patients are mostly affected in childhood or young adults. Intending to minimize the treatment burden on patients, the identification of biomarkers for treatment stratification and evaluation of treatment response is of critical importance. Recently, tumor cell content in germinomas has been shown to be closely related to prognosis, suggesting that cases with low tumor cell content may be safely treated with a less intensive regimen. Among the copy number alterations, the 12p gain is the most prominent and has been shown to be a negative prognostic factor in NGGCTs. MicroRNA clusters (mir-371-373) were also revealed to be a hallmark of GCTs, demonstrating the potential for the application of liquid biopsy in the diagnosis and detection of recurrence. Recurrent mutations have been detected in the MAPK or PI3K pathways, most typically in *KIT* and *MTOR* and low genome-wide methylation has been demonstrated in germinoma; this most likely reflects the cell-of-origin primordial germ cells for this tumor type. These alterations can also be leveraged for liquid biopsies of cell-free DNA and may potentially be targeted for treatment in the future. Advancements in basic research will be translated into clinical practice and can directly impact patient management. Additional understanding of the biology and pathogenesis of GCTs will lead to the development of better-stratified clinical trials, ultimately resulting in improved treatment outcomes and a reduction in long-term treatment-related adverse effects.

## Introduction

Central nervous system (CNS) germ cell tumors (GCTs) mostly occur in adolescents and young adults (AYA), with a median age of onset of 12–16 years (1–3). These tumors have a male predominance, with an estimated male-to-female ratio of 8:2; however, this depends on the site of occurrence. The pineal region is the most frequent location for GCTs, followed by the neurohypophysis, lateral ventricles, basal ganglia, cerebral and cerebellar hemispheres, and brainstem. CNS GCTs are more common in East Asia than in Western countries, and the site of occurrence differs geographically; tumor locations at the basal ganglia are more common in East Asia (4). Histopathologically, GCTs are mainly categorized into germinomas and non-germinomatous GCTs (NGGCTs); NGGCTs can be categorized as teratomas, yolk sac tumors, choriocarcinomas, and embryonal carcinomas (5). Teratomas include mature, immature, and teratoma with somatic-type malignancy. GCTs often present as a mixture of these components. Treatment is based on a combination of surgery, chemotherapy, and radiation therapy. GCTs are known to be sensitive to chemotherapy and radiation therapy (except for mature teratomas). The initial role of surgery in the treatment course is to obtain specimens for diagnosis, especially in cases presenting with negative tumor markers (i.e., human chorionic gonadotropin [HCG] and alpha-fetoprotein [AFP]). A biopsy is often omitted when these markers are elevated, along with imaging and clinical presentations compatible with GCTs. However, remnant tissue after chemotherapy and/or radiation therapy requires resection to decrease the chance of future enlargement and for diagnosis.

Treatment is mainly stratified according to the histological group, whether the tumor is a germinoma or NGGCT. The exceptions to this are the abovementioned mature teratomas, which only require surgical resection. Germinomas are treated less intensively than NGGCTs because the former tumor type is more sensitive to available therapies. In Europe and North America, cases with typical presentations in terms of imaging findings and clinical backgrounds undergo tumor marker examination. Tumor markers (HCG and AFP) are examined in blood serum and cerebrospinal fluid (CSF) from lumbar puncture or ventricular drainage when it is safe to obtain the sample for the latter. If tumor markers are elevated, patients are diagnosed with NGGCTs; if not, patients require a biopsy for histopathological diagnosis (6–10). In Japan, treatment stratification is unique in that it is based on three prognostic subgroups (i.e., germinoma, intermediate prognosis, and poor prognosis subgroups) that are determined according to histopathological confirmation (**Table 1**) (11, 12).

**Table 1:** Difference in diagnosis and risk stratification among the US, Europe, and Japan.

	Germinoma	NGGCT	
COGACNS1123	Pathology-based; HCG≤100IU/I and AFP≤10ng/ml	Tumor marker-based; HCG>100IU/I or AFP>10ng/ml OR Pathological diagnosis if markers are negative	
SIOP GCTII	Pathology-based; HCG≤50IU/I and AFP≤25ng/ml	Tumor marker-based; HCG>50IU/I or AFP>25ng/ml OR Pathological diagnosis if markers are negative	
Japan	Pathology-based	Intermediate prognosis group	Poor prognosis group
		Pathology-based; Immature teratoma, teratoma with somatic-type malignancy, mixed GCTs mainly composed of germinoma and teratoma	Pathology-based; choriocarcinoma, embryonal carcinoma, yolk sac tumor, mixed GCTs mainly composed of these components

One of the ongoing issues in treatment and patient care is that treatment stratification is currently mostly limited to two or three categories and predictive biomarkers for guiding personalized treatment are presently scarce. As previously noted, CNS GCTs predominantly occur in the pediatric, adolescent, and young adult (AYA) population. The profound effects of chemotherapy and radiation therapy on pediatric and young adult patients cannot be overlooked. Secondary malignancies, including gliomas and melanomas, cavernous angiomas, meningiomas, vascular constrictions, and brain atrophy, are only a few of the concerns (13–18). Clinical trials are mainly concerned with the reduction of treatment burden while obtaining similar treatment outcomes; risk stratification has not yet been established.

However, recent clinical and molecular investigations present new key findings that may have prognostic or predictive value. Here, we present candidate biomarkers that have been discovered in research conducted on a global scale.

## Germinoma

### Treatment overview

There is a scientific and medical consensus that the most appropriate treatment for germinomas occurring at typical sites, including the neurohypophysis, pineal gland, and lateral ventricles, is whole ventricular irradiation (WVI, with or without a local irradiation boost) and platinum-based chemotherapy (19). Upfront surgery is reserved for obtaining tumor biopsies for histopathological diagnosis. Historically, clinical trials conducted worldwide have proven that local irradiation is insufficient and that relapses occur (these relapses are mostly restricted to the ventricular system, with rare occurrences in the spinal canal) (7, 20, 21). The SIOP CNS GCT-96 trial demonstrated a 5-year progression-free survival (PFS) of 97 ± 2% in patients treated with craniospinal irradiation (CSI) and a PFS of 88 ± 4% in patients treated with local irradiation (7). COG ACNS1123 (stratum 2) showed a three-year PFS of 94% in patients treated with WVI at 18 Gy plus local irradiation at 12 Gy (**Table 2**) (22).

**Table 2:** Representative clinical trials for germinomas across countries.

Region	North America		Europe		Japan	
Name of clinical trial	COG ACNS0232	COG ACNS1123 (stratum 2)	SIOP CNS GCT-96	SIOP CNS GCTII	Multi-institutional prospective study of iGCTs funded by Ministry of Health, Labour and Welfare	Phase II clinical study of chemotherapy and radiation therapy for patietns with primary intracranial germ celltumors
Time period	2007-2009	2012-2016	1996-2005	2012-2018	1995-2003	2010-
Number of cases	24	137	235	Unknown	123	155
Eligibility	Any	Localized tumor	Any	Any	Any	Any
Radiation therapy	WVI 24Gy+LRT 21Gy (w/o chemotherapy) OR LRT 30Gy (w/ chemotherapy and CR)	WVI 18Gy/12Fr+LRT 12Gy/8Fr (CRto chemotherapy or no malignancy at second-look surgery)	CSI 24Gy/15Fr + LRT 16Gy/10Fr (w/o chemotherapy) OR LRT 40Gy (w/ chemotherapy) (Localized)	WVI 24Gy/15Fr (Localized, CR to chemotherapy) WVI 24Gy/15Fr+LRT 16Gy/10Fr(Locaized, non-CR to chemotherapy)	Extended LRT24Gy/12Fr	WVI 23.4Gy/13Fr (Localized) CSI23.4Gy/15Fr (Disseminated)
		WVI 24Gy/16Fr+LRT 12Gy/8Fr (Non-CRto chemotherapy and normal marker and no second-look surgery)	CSI 24Gy/15Fr+LRT 16Gy/10Fr (w/ORw/o chemotherapy) (Disseminated)	WVI 24Gy/15Fr+LRT 30.4Gy/19Fr(Locaized, SD and incomplete resection at second-look surgery) CSI 24Gy/15Fr + LRT 16Gy/10Fr+focal irradiation to met 16Gy/10Fr (Disseminated)		
Chemotherapy	Carboplatin + Etoposide	Carboplatin 600mg/sqm + Etoposide 450mg/sqm 4 cycles	Carboplatin 600mg/sqm + Etoposide 300mg/sqm with Ifosfamide 9000mg/sqm + Etoposide 300mg/sqm ('CarboPEI') 2 cylces	Carboplatin 600mg/sqm + Etoposide 300mg/sqm with Ifosfamide 9000mg/sqm + Etoposide 300mg/sqm ('CarboPEI') 2 cycles	Carboplatin 450mg/sqm + Etoposide 450mg/sqm ('CARE') 3 cycles	Carboplatin 450mg/sqm + Etoposide 450mg/sqm ('CARE') 3 cycles
Outcome	Not published (early study closure)	3yPFS 94±3% (WVI 18Gy+LRT12Gy)	5yPFS 97±2% (CSI), 5yPFS 88±4% (LRT) (Localized), 5yPFS 100% (Disseminated)	Not published yet	5yPFS: 88%, 5yOS: 98%	During accrual

WVI, whole ventricular irradiation; LRT, local radiation therapy; CSI, cranio-spinal irradiation; met, metastasis; CR, complete response; PR, partial response; Fr, fraction; PFS, progression-free survival; OS, overall survival.

An issue of concern is that the treatment strategy is currently homogeneous, without any risk stratification, except for whether one is addressing localized or disseminated disease. Now that the treatment protocol has matured (although without stratification), researchers should endeavor to find clues for identifying cases where the treatment burden can be reduced. Moreover, although the treatment outcome for this tumor type is good, with a 5-year PFS of approximately 90%, the remaining 10% of patients experience treatment failure. Researchers should aim to identify the features that characterize those failure cases at the initial presentation.

### Tumor markers

Some germinoma cases present with elevated HCG levels in the serum and/or cerebrospinal fluid (CSF), and there are still controversies over the prognostic significance of elevated HCG in germinoma. The so-called “HCG-producing germinoma” is sometimes reflected by the presence of syncytiotrophoblastic giant cells (STGCs) in histopathological specimens, which indicates strong immuno-positivity for HCG. In a previous study of 74 histologically confirmed germinoma cases, 33 (45%) showed a higher than normal cut-off value for serum HCG, and those cases did not present worse relapse-free survival (RFS) than those with normal HCG values (23). In contrast, a study carrying out a highly sensitive measurement of HCG in CSF demonstrated that >85% of germinoma cases showed elevated CSF levels and that those with highly elevated HCG levels in the CSF (9/35 cases) showed worse RFS than those with lower HCG levels (24). Of note, regarding messenger RNA (mRNA) expression levels, almost all germinomas are known to express HCG mRNA (25). Collectively, although germinomas inherently produce HCG at baseline, highly elevated HCG in the CSF (with an undetermined cut-off line) seems to be associated with an unfavorable prognosis; these tumors should be carefully monitored for recurrence or dissemination on follow-up.

### Atypical site germinoma

The sites of germinoma occurrence are predominantly at the midline of the brain, including the pineal gland, neurohypophysis, and lateral ventricles (and combinations thereof, i.e., typical sites). However, germinomas occasionally occur in the basal ganglia, thalamus, cortex/subcortex of the cerebrum, cerebellum, and spinal cord (i.e., atypical sites). Previous studies have revealed that cases with tumors occurring at atypical sites, such as those described above, show a poor prognosis compared with those occurring at typical sites (1, 26)., This may be due to the non-standard treatment regimens associated with atypical tumors. More specifically, typical site germinoma can be treated with WVI (with or without local irradiation). However, cases at atypical sites have to be managed differently and no appropriate radiation coverage has been elucidated based on clinical trials. Many clinical trials exclude germinomas at atypical sites, partly because these cases rarely occur, especially in Western countries (4).

We note that most of these cases should be treated with whole-brain or craniospinal irradiation. In a retrospective study, cases with basal ganglia germinoma treated with whole-brain or craniospinal irradiation showed better RFS than those treated with focal irradiation or WVI (26). Another potential reason for prognostic differences across tumor types includes biological differences between these tumors; for example, a prior study demonstrated that germinomas occurring at atypical sites had more frequent mutations in the PI3K pathway than those occurring at other sites (1). Atypical-site germinomas may also be more aggressive in nature. Conducting a large-scale study to determine the best management regimen is challenging because of the low frequency of this tumor outside of Asian countries. Future clinical trials should focus on the best radiation coverage and dosing strategies and investigate whether an adjustment in chemotherapy intensity could reduce patients’ radiation treatment burden.

### Immune cell infiltrates

Germinoma has well-known histopathological characteristics, with a germinoma cell and immune cell mixture that is dubbed a “two-cell pattern” (27). Most dominant immune cells are lymphocytes (T-cell and B-cell, approximately 60-70% on average) and natural killer cells, but immune cell repertoire in germinoma also includes myeloid-derived cells such as macrophages accounting for approximately 20-30% on average (28). The ratio between germinoma cells and immune cells is known to vary substantially depending on case characteristics, with <10% to 90% of tumor cells (out of all nucleated cells in tissue specimens) observed on hematoxylin-eosin (H-E) staining (29). As germinoma cells are obviously larger than immune cells and their cell morphologies differ, it would not be very challenging to calculate the ratio of tumor cells to immune cells in daily clinical practice. One of the novel aspects of evaluating and stratifying germinoma treatment strategies is that cases with many immune cells have shown better prognoses than those with a high tumor content in previous research (28). Moreover, this finding remained statistically significant when incorporating information on tumor location, as tumor location is another prognosis-related factor, as described previously (29). Currently, it is not standard practice to only treat germinoma with chemotherapy without radiation therapy, based on the results of previous clinical trials in which frequent relapses were observed in patients with germinoma treated with chemotherapy only (30). Conversely, a study evaluating chemotherapy-only regimens in 13 germinoma cases found that approximately 20–30% of these patients did well; that is, they did not experience recurrence within 5 years (30). Thus, a certain proportion of germinoma cases may be cured with chemotherapy alone. Germinoma presenting with abundant immune cells may be a good candidate for reduced treatment intensity (29).

Biological markers should be comprehensively evaluated in future clinical trials aiming to reduce the radiation dose administered to germinoma cases with abundant immune cells, whether regarding tumor content in H-E-stained specimens or other markers. A recent study revealed that germinoma cases could be subdivided into two subgroups when integrating transcriptome and methylome data (31); this stratification was reflected by differentiating the tumor cell-immune cell ratio. Additional studies are warranted to comprehensively and reliably identify the germinoma subgroup, which can be treated less intensively, and to identify relevant biomarkers.

## Non-germinomatous germ cell tumors

### Treatment overview

In contrast to treatment for germinoma, the treatment for NGGCT has not yet been standardized worldwide. First, classifications differ in Europe, North America, and Japan. In Europe/North America, NGGCT is defined according to a case presentation in which tumor markers are elevated or according to histopathological confirmation of NGGCTs when tumor markers are normal (10). In Japan, NGGCT is further subdivided into two classes: an intermediate prognosis group and a poor prognosis group (3, 11)., The intermediate prognosis group includes immature teratomas, teratomas with somatic-type malignancy, and mixed tumors (mainly composed of germinomas and teratomas). The poor prognosis group mainly includes patients with yolk sac tumors, choriocarcinomas, and embryonal carcinomas. These two groups were treated differently in terms of radiation coverage and the selected chemotherapy regimen in previous research (3). Moreover, we note that tumor marker cut-off values differed between SIOP (HCG >50 IU/L, AFP 25 ng/mL) and COG (HCG >100 IU/L, AFP 10 ng/mL) clinical trials, which led to differences in the patients that were registered as NGGCT cases in these trials. These differences in categorization make it difficult to compare treatment outcomes across studies conducted in the three abovementioned regions.

Localized NGGCTs were treated with WVI and local irradiation in COG ACNS 1123 (8), and are presently treated with WVI and spinal irradiation in ACNS 2021 (an ongoing study in North America). In Europe, NGGCTs have been treated with local irradiation in SIOP CNS GCT-96 and CNS GCTII (6, 7). In Japan, NGGCTs are treated with WVI and local irradiation in the intermediate prognosis group, whereas NGGCTs are treated using CSI and local irradiation in the poor prognosis group; these treatments are selected as a matter of protocol.3 Cases with multiple lesions, except for bifocal (neurohypophysis and pineal gland) lesions, are universally treated with CSI (**Table 3**).

**Table 3:** Representative clinical trials for non-germinomatous germ cell tumors (NGGCTs) across countries.

Region	North America			Europe		Japan	
Name of clinical trial	COG ACNS0122	COG ACNS1123(stratum 1)	COG ACNS2021	SIOP CNS GCT-96	SIOP CNS GCTII	Multi-institutional prospective study of iGCTs funded by Ministry of Health, Labour and Welfare	Phase II clinical study of chemotherapy and radiation therapy for patietns with primary intracranial germ cell tumors
**Time period**	2004-2008	2012-2016	2021-	1996-2005	2012-2018	1995-2003	2010-
Number of cases	102	107	160 (estimated)	149	Unknown	83(lntermediate), 27(Poor)	35(lntermediate), 20(Poor) (estimated)
Eligibility	Any	Localized tumor	Localized tumor (SS and/or pineal)	Any	Localized disease	Any	Any
Radiation therapy	CSI 36Gy/24Fr+LRT 18Gy/12Fr (CR/PRto chemotherapy w/ or w/o second-look surgery)	WVI 30.6Gy/17Fr+LRT 23.4Gy/13Fr (CR/PR to chemotherapy w/ or w/o second-look surgery)	WVI + SCI30.6Gy/17Fr +LRT23.4Gy/13Fr (CR/PR to chemotherapy and normal marker OR MT/non-viable tumor on second-look surgery with normal marker)	LRT 54Gy/30Fr (Localized)	LRT 54Gy/30Fr (Localized)	Extended LRT 30Gy/15Fr+LRT 20Gy/10Fr (Intermediate)	WVRT 23.4Gy/13Fr + LRT27Gy/15Fr (Intermediate)
			CSI 36Gy/24Fr+LRT 18Gy/12Fr (not CR/PR and not PD)	CSI 30Gy/20Fr+LRT 24Gy/15Fr (Disseminated)	CSI 30Gy/20Fr + LRT 24Gy/15Fr+Spinal LRT 20.8Gy/13Fr (Disseminated)	CSI 30Gy/15Fr+LRT 30Gy/15Fr(Poor)	CSI 30.6Gy/17Fr+LRT 30.6Gy/17Fr(Poor)
Chemotherapy	Carboplatin 600mg/sqm + Etoposide 270mg/sqm with Ifosfamide 9000 mg/sqm + Etoposide 450 mg/sqm 3 cycles each plus Thiotepa 900mg/sqm + 1500mg/sqm and PSCR (not CR/PR)	Carboplatin 600mg/sqm + Etoposide 450mg/sqm with Ifosfamide 9000 mg/sqm + Etoposide 500 mg/sqm 3 cycles each	Carboplatin + Etoposide with Ifosfamide + Etoposide 3 cycles each plus Thiotepa + Etoposide and PSCR (not CR/PR)	Cisplatin 100mg/sqm + Etoposide 300mg/sqm + Ifosfamide 7500mg/sqm ('PEI') 4 cycles	Cisplatin 100mg/sqm + Etoposide 300mg/sqm + Ifosfamide 7500mg/sqm ('PEI') 4 cycles (Standard risk)	Carboplatin 450mg/sqm + Etoposide 450mg/sqm ('CARE') 8 cycles (Intermediate)	Carboplatin 450mg/sqm + Etoposide 450mg/sqm ('CARE') 3 cycles (Intermediate)
					Cisplatin 100mg/sqm + Etoposide 1500mg/sqm + Ifosfamide 10000mg/sqm ('HyperPEI') + ASCT (High risk)	Ifosfamide 4500mg/sqm + Cisplatin 100mg/sqm + Etoposide 300mg/sqm ('ICE') 8 cycles (Poor)	Ifosfamide 4500mg/sqm + Cisplatin 100mg/sqm + Etoposide 300mg/sqm ('ICE') 8 cycles (Poor)
Outcome	5yEFS: 84±4%, 5yOS: 93±3%	3yPFS: 88±4%, 3yOS: 92±3% (early study closure due to spinal relapse)	During accrual	5yPFS: 72±4%, 5yOS: 82±4% (Localized), 5yPFS: 68±9%, 5yOS: 75±8% (Disseminated)	Not published yet	5yPFS: 85%, 5yOS: 95% (Intermediate), 5yPFS: 59%, 5yOS: 59% (Poor)	During accrual

WVI, whole ventricular irradiation; LRT, local radiation therapy; CSI, cranio-spinal irradiation; SCI, spinal canal irradiation; PSCR, peripheral stem cell rescue; ASCT, autologous stem cell transplantation; CR, complete response; PR, partial response; Fr, fraction; PFS, progression-free survival; OS, overall survival.

### Age

The SIOP GCTII study identified patients <6 years old as a high-risk subgroup among patients with NGGCT (6). This coincides with the biological categorization of GCTs into seven classes of whole-body GCTs, where cases <6 years old are categorized as type I (in contrast to many CNS cases that fall into the type II category) (32). Most type I cases comprise yolk sac tumors, teratomas, and their combination. These cases are clinically aggressive and require intensive treatment with high-dose chemotherapy and autologous stem cell rescue.

### Tumor markers

The SIOP GCTII study identified an AFP level of >1,000 ng/mL as a poor prognostic factor (6) The fraction of these cases was small (19/149, 12.8%); however, this subgroup showed a worse prognosis, with a higher relapse rate. This strongly suggests that patients with yolk sac tumors or yolk sac tumor components have a relatively poor prognosis among the overall population of those with NGGCTs. This finding needs to be validated in a larger cohort. We note that Japanese clinical trials have determined that patients with HCG levels of >2,000 IU/L or AFP levels of >2,000 ng/mL are eligible for upfront chemotherapy and radiation therapy without histopathological confirmation.

### Residual mass after chemotherapy

Residual masses are often found after chemotherapy +/- irradiation. The presence of a malignant component at histopathological confirmation following chemotherapy helps in stratifying the subsequent treatment regimen. More specifically, the existence of a residual mass has not by itself been shown to have prognostic significance. However, residual masses, with or without second-look surgery, have been associated with a higher relapse rate (6). Interestingly, most residual masses detected after chemotherapy and radiation therapy comprise teratomas and/or necrotic tissue (17, 33). In COG ACNS2021, if viable tumor cells are confirmed in resected specimens at second-look surgery following radiation therapy, high-dose chemotherapy with peripheral stem cell rescue is administered.

### 12p gain

GCTs are chromosomally unstable tumors with abundant gains and losses of chromosomal arms (34). Among these, 12p gain is one of the most frequent copy number alterations found in approximately 40% of cases. In contrast, 12p gain is more common in testicular GCTs and is found in up to 90% of these tumors (35). A study on 12p gains in CNS GCTs found that this gain was considerably more common in patients with NGGCTs as compared with patients with germinomas and was especially frequent in patients with NGGCTs accompanied by malignant components (including yolk sac tumors, choriocarcinomas, and embryonal carcinomas). Patients with NGGCTs presenting with a 12p gain have been shown to have a shorter PFS and OS than those without the 12p gain. Moreover, this previous study showed that 12p copy number status was shared among different histological components (i.e., across germinoma and non-germinoma components) in the same tissue specimen when examined by microdissection and fluorescence in situ hybridization (FISH) analysis (36). We note that KRAS is an oncogene in this genomic region that is known to exhibit a pathognomonic mutation in GCTs (37); 12p gain was found to be mutually exclusive with the most frequent mutation in CNS GCTs, KIT mutation (36). 12p gain may serve as a biological marker for poor prognosis, which can be investigated on copy number analysis based on a methylation array or on FISH analysis.

### 3p25.3 gain

A recent study in patients with testicular GCTs found that 3p25.3 gain was a poor prognostic factor among patients with non-seminomatous GCTs (38). This gain was present in 6.8% of cases, and those that harbored this gain showed shorter PFS and OS than those without. This gain has also been observed in a few seminoma cases and has been associated with resistance to platinum-based chemotherapy. This study did not evaluate specific genes in this genomic region that may potentially be involved in these biologically and clinically significant findings.

The frequency of this copy number alteration in CNS GCT populations has not yet been investigated. A study on the biological and clinical significance of the 3p25.3 gain among CNS counterparts is needed.

## Omics analysis

According to a study conducting exome sequencing and targeted sequencing in 124 CNS GCTs, mutations in the MAPK pathway (more precisely, the KIT/RAS pathway) and the PI3K pathway were found in 48% and 13% of the evaluated cases, respectively (37). In total, 55% of the analyzed cases exhibited at least one of these mutations. Mutations were found more frequently in germinoma cases (71%) than in NGGCT cases (34%). The KIT mutation was the most predominant. Moreover, regardless of the mutation type, all germinoma cases were immunopositive for the KIT protein, which suggests that high expression of KIT is a marker for germinoma; this corroborates the fact that migrating primordial germ cells (PGCs), which are hypothesized to be the cell-of-origin for this cancer type, depend on the KIT-KIT ligand for proliferation and survival (39). KIT is a receptor tyrosine kinase (RTK) recognized by antibodies to CD117 (c-Kit). KIT inhibitors are novel therapeutic agents that require testing in international clinical trials. Non-specific oral KIT inhibitors exist in the clinic, but of those RTK inhibitors in general use, dasatinib but not imatinib reliably crosses the blood-brain barrier (40). Inhibitors of mTOR, MEK, and ERK may also potentially play a role in the pathogenesis of GCTs.

Genome-wide methylation array analysis has shown that germinomas are characterized by strikingly low levels of methylation across the genome (41, 42). Hypomethylation may render GCTs unstable on a chromosome and genome level, making these tumors prone to progression. Notably, hypomethylation is observed in retrotransposons, such as LINE-1 and Alu; hypomethylation can activate retrotransposons in the genome, thereby leading to genomic structural abnormalities.

Transcriptome analysis by RNA sequencing has demonstrated that germinoma is characterized by a high expression of PGC- and meiosis-related marker genes, and that NGGCT is characterized by a high expression of genes related to tissue/organ development, Wnt/beta-catenin pathway signaling, and epithelial-mesenchymal transition (31) (43). These features were corroborated by protein expression findings in immunohistochemistry, where pluripotency markers (including KIT, Oct3/4, NANOG, TFAP2c) and germ cell markers (including MAGEA4, NY-ESO-1, TSPY) were highly positive in germinomas (44).

Moreover, comparative studies evaluating CNS and testicular GCTs showed that germinomas and seminomas, as well as NGGCTs and non-seminomatous GCTs (NSGCTs), were grouped together on methylation analysis. While there were differences in expression between germinomas and seminomas, NGGCTs and NSGCTs were clustered on expression analysis (31). This suggests that the cell-of-origin for GCTs is shared across GCTs of different origins and that these tumor types might share similar pathogenesis. We note that studies on new biomarkers and novel therapeutic agents may be possible using shared platforms across different organs.

These omics analyses have clarified many biological aspects of CNS GCTs. Although the development of targeted therapies and the identification of clinically useful biomarkers based on these findings is still under-investigated, this field has considerable potential.

## MicroRNAs

MicroRNAs (miRNAs) derived from miR-371a-373p, and miR-302/367 clusters have been implicated in the pathogenesis of GCTs. These miRNAs are consistently expressed in germinomas, and TP53 is suspected to be functionally inactivated by miRNAs arising from the miR-371a-373p cluster (45). These biomarkers were reported to outperform standard tumor markers in terms of sensitivity and specificity for detecting GCTs of the testis in a prospective study (46). For CNS GCTs, their roles would include early detection of GCTs (before the appearance on imaging) and early detection of tumor recurrence during and after treatment (47). Prospective studies conducted in a large cohort, including various histological subtypes, are warranted to elucidate the function of these miRNAs in CNS GCTs. Moreover, because of their suspected involvement in the TP53-MDM2 signaling pathway, a targeted treatment approach may be applicable in reference to these biomolecules.

## Conclusion

The past decade has seen reports of a few key clinical trials originating from Europe and North America. Based on these findings, new clinical trials that are necessary to further elucidate the appropriate treatment intensities for both germinomas and NGGCTs have been started. Alongside this, basic research (including several omics studies) has been ongoing; this research has clarified important biological aspects of GCTs (**Table 4**). However, risk stratification is still underdeveloped as compared with that for other pediatric brain tumors. Additional efforts are needed to investigate potential risk factors, and clinical trials examining how to incorporate these factors into clinical practice in order to adjust treatment intensity are necessary. Because GCT is primarily a pediatric and adolescent disease, timely studies informing the reduction of long-term treatment-related sequelae and the resulting clinical implementation of these evidence-based findings are of critical importance.

**Table 4:** Current and developing biomarkers for CNS GCTs.

Biomarkers	Tumor marker	Tumor cell content	12p gain	3p25.3 gain	MicroRNAs
Specimen	Blood serum & CSF	H-E pathological slides	DNA (for methylation), FFPE for FISH	DNA (for methylation)	DNA
Advantages	Routinely examined, able to differentiate germinoma and NGGCTs, able to detect recurrence	Cases with high tumor cell content show poorer prognosis in germinoma	Differentiate germinoma and NGGCTs, identifies malignant NGGCTs	May identify malignant NGGCTs	Better sensitivity and specificity than tumor markers in detecting GCTs
Disadvantages	Not necessarily sensitive nor specific	Not established; needs validation	Not established; needs validation	Not examined in CNS GCTs	Not able to differentiate germinoma and NGGCTs, not fully validated in CNS GCTs

CSF, cerebrospinal fluid; H-E, hematoxylin and eosin; FFPE, formalin-fixed paraffin-embedded; FISH, fluorescent in-situ hybridization; NGGCT, non-germinomatous germ cell tumor; GCT, germ cell tumor.

## Author contributions

HT designed and wrote the manuscript. KI wrote and checked the manuscript. All authors contributed to the article and approved the submitted version.

## Funding

This study was partially sponsored by the Japanese Foundation for Multidisciplinary Treatment of Cancer (JFMC2021; no grant number) and Grant-in-Aid for Young Scientists, KAKENHI No. 22K16650 from the Japan Society for the Promotion of Science (JSPS).

## Conflict of interest

The authors declare that the research was conducted in the absence of any commercial or financial relationships that could be construed as a potential conflict of interest.

## Publisher’s note

All claims expressed in this article are solely those of the authors and do not necessarily represent those of their affiliated organizations, or those of the publisher, the editors and the reviewers. Any product that may be evaluated in this article, or claim that may be made by its manufacturer, is not guaranteed or endorsed by the publisher.
